# Prevalence, pattern of disease and antimicrobial susceptibility of *Candidozyma auris* in the greater Pretoria region from 2021 to 2024

**DOI:** 10.4102/sajid.v41i1.817

**Published:** 2026-05-06

**Authors:** Abdullah Moola, Marko G. Thom, Jonah M. Finlay, Roxanne Rule, Mohamed Said

**Affiliations:** 1 Faculty of Health Sciences, University of Pretoria, Pretoria, South Africa; 2 Department of Medical Microbiology, University of Pretoria, Pretoria, South Africa; 3 National Health Laboratory Service, Tshwane Academic Division, Tshwane, South Africa

**Keywords:** *Candidozyma auris*, susceptibility, prevalence, South Africa, Pretoria, fungal disease, candidaemia, *Candida auris*

## Abstract

**Background:**

*Candidozyma auris* has emerged as a nosocomial pathogen in South Africa, characterised by multidrug resistance and environmental persistence.

**Objectives:**

This study aimed to describe the prevalence, disease patterns, and antifungal susceptibility patterns of *C. auris* isolates recovered from public-sector healthcare facilities in the greater Pretoria region from 2021 to 2024.

**Method:**

A retrospective laboratory-based surveillance study was conducted using data from the National Health Laboratory Service Tshwane Academic Division laboratory. Isolates were classified as invasive or non-invasive based on specimen source. Temporal trends in antifungal minimum inhibitory concentrations (MICs) were analysed using interval-censored regression.

**Results:**

A total of 592 *C. auris* isolates were identified. Blood cultures were the most frequent specimen source overall, comprising 237 isolates (40.03%). Intravascular catheter tip isolates predominated in 2023 and 2024, with 48 and 72 isolates, respectively. The proportion of invasive isolates declined from 56.8% to 40.8% over the study period. Among tested isolates, fluconazole resistance exceeded 99%. Resistance to amphotericin B and echinocandins was uncommon, with eight total isolates identified. Decreasing MIC trends were observed for amphotericin B (β = −0.059 per year; *p* = 0.012) and micafungin (β = −0.081 per year; *p* = 0.026).

**Conclusion:**

*Candidozyma auris* remains established in the public-sector within the greater Pretoria region. There is a shift from invasive bloodstream infections towards non-invasive, device-associated isolates. Fluconazole resistance remained high while amphotericin B and echinocandins retained good *in vitro* activity.

**Contribution:**

This study contributes to the knowledge of *C. auris* in the greater Pretoria region, providing insight into epidemiology and antifungal susceptibility.

## Introduction

Fungal diseases kill more than 1.5 million people worldwide and infect over a billion more.^1^ Despite most fungal diseases being avoidable, they remain a neglected topic by public health officials.^1^ Furthermore, invasive fungal infections are increasingly acknowledged as a significant concern in critically ill adult and paediatric patients.^2^ South Africa also has a high burden of fungal disease, generally as a secondary infection in patients already infected with human immunodeficiency virus (HIV) and acquired immunodeficiency syndrome (AIDS), tuberculosis (TB) or groups with low socioeconomic status.^3^ *Candida albicans* has historically been the predominant cause of candidiasis and candidaemia, but over the past decade, there has been a global shift towards non-albicans fungal species.^4^ One of these emerging species in South Africa is *Candidozyma auris*, and it affects both the private and public sectors.^5^

According to global estimates compiled from published studies by the Global Action Fund for Fungal Infections (GAFFI), *C. auris* ranks as the fifth most common life-threatening fungal infection and carries an estimated mortality rate of 40%.^1^ In South Africa, the incidence of candidaemia has been reported at 83.8 cases per 100 000 hospital admissions, with rates varying by hospital and fungal species.^5^ National surveillance from 2016 to 2017 indicated that *C. auris* accounts for 10% of all candidaemia cases in South Africa.^6^ Infections occur in both immunocompetent and immunocompromised patients, with a higher incidence in immunocompromised individuals. Candidiasis has been referred to as the ‘disease of diseased’.^4^

The factors associated with the highest risk of invasive fungal infections are broad-spectrum antibiotics, blood transfusions, central venous catheters, *Candida* colonisation, and total parenteral nutrition.^7^ Comorbid health conditions, such as HIV infection and diabetes mellitus, also increase the risk of developing an invasive fungal infection.^7^

*Candidozyma auris* was first reported in Japan in 2009.^8^ The first case in South Africa also appeared in 2009, but it was misidentified as *Candida haemulonii* and was only correctly identified retrospectively in 2014.^6^

*Candidozyma auris* is an emerging pathogen that is rapidly increasing in prevalence both locally and internationally. In 2016, there were 1692 confirmed or probable cases of *C. auris*, and by 2017, *C. auris* was responsible for 10% of candidaemia cases in South Africa.^5,6^ The global mortality rate of *C. auris* is estimated at 39%, with regional mortality rates reaching as high as 75%. This demonstrates the danger of this emerging disease.^9^

*Candidozyma auris* is a germ tube-negative yeast and is able to grow at relatively high temperatures (42 °C).^10^

While it does appear pink or purple on chromogenic *Candida* agar, molecular identification is the reference standard method for species-level identification.^11,12,13^ However, this is not readily available in routine diagnostic labs, and diagnosis depends on culture followed by identification using matrix-assisted desorption ionisation-time of flight (MALDI-TOF).^14^

*Candidozyma auris* clinical infection presents in a similar manner to that of other fungal species.^15^ Sites from which *C. auris* has been isolated include the respiratory tract, the heart and liver, blood, the abdominal cavity, rectal or stool culture, urine, vagina, bone, axilla, groin, wounds or surgical tissue, pus, ear, and brain.^15^ Reported infections include fungaemia, myocarditis, urinary tract infection, surgical wound infections, burn infections, skin abscesses, otitis, meningitis, and bone infections.^15^

There are three classes of systemic antifungals commonly available for the treatment of invasive fungal infections, namely the polyenes, triazoles, and echinocandins. *Candidozyma auris* was found to be resistant to two of the classes in 41% of cases and resistant to all three in 4% of cases in a 2016 surveillance study that spanned three continents, including samples from South Africa.^16^ In South Africa, *C. auris* has consistently high fluconazole minimum inhibitory concentration (MIC) values and occasionally high amphotericin B and echinocandin MICs.^8,14,17^

*Candidozyma auris* has been shown to contaminate the hospital environment and equipment for prolonged periods of time.^18,19^ It can also colonise patients admitted to intensive care units at multiple body sites such as the axilla, rectum, groin and oral cavity.^18,19^ Daily cleaning of the patient’s environment should be performed using a neutral detergent and water, thereafter the surfaces should be wiped with 1000 parts per million sodium hypochlorite solution. Alternative disinfectants such as quaternary ammonium compounds and ethyl alcohol have demonstrated reduced efficacy and are not recommended.^14^

Given the increasing incidence of clinically relevant disease presentation and the high mortality in invasive disease, accurate information about disease patterns is important in public health planning. This study investigated the prevalence of *C. auris* in the greater Pretoria area, its antifungal susceptibility patterns, and the most common specimen sources of *C. auris* infections in this population group.

## Research methods and design

### Study design

A retrospective laboratory-based surveillance study was performed from January 2021 to December 2024 using Microbiology Laboratory data provided by the Tshwane Academic Division (TAD) Microbiology laboratory.

### Setting

This study was conducted in the greater Pretoria area using information from Steve Biko Academic Hospital, Tshwane District Hospital, Pretoria West Hospital, Kalafong Provincial Tertiary Hospital, and Tembisa Provincial Tertiary Hospital as well as many other smaller clinics in the area that are serviced by the National Health Laboratory Services (NHLS) TAD Microbiology laboratory. This study only covers the public health system and does not have any data from private healthcare facilities.

The TAD Microbiology laboratory is responsible for processing all microbiological cultures for this area and documents each occurrence of *C. auris*.

### Study sample

All patients from whom a clinical specimen cultured *C. auris* between January 2021 and December 2024 were included in the study. The database was then de-duplicated to remove multiple repeat isolates from the same patient within a 3-week period, as per guidance provided in the Clinical and Laboratory Standards Institute M39 document.^20^ In such cases of de-duplication, blood cultures were prioritised as the recorded source of isolation as this was considered an indication of an invasive infection. It is important to observe that susceptibility testing was referred to another laboratory during the study period as the TAD laboratory did not have standardised testing available. The methodology used by the referral laboratory was the Sensitire YeastOne YO10 AST Plate (Thermofisher, United States [US]).

### Data collection

Laboratory data were extracted from the NHLS Corporate Data Warehouse (CDW). The extracted data contained the age, gender, hospital location, ward number and antifungal susceptibility results of the isolates. Clinical outcome could not be determined because it was not recorded on the NHLS CDW.

### Data and statistical analysis

Initial data analysis was performed in Microsoft Excel. For isolate distribution, proportions of invasive versus non-invasive isolates were compared across years using a chi-square test for independence, and a linear regression model was fitted to assess the presence and direction of a linear trend in the proportion of invasive isolates over time. The chi-square test evaluated overall year-to-year differences, while the regression coefficient for year quantified the annual change in invasiveness (β < 0 indicating a downward trend). A two-sided *p* < 0.05 was considered statistically significant.

For the antifungal susceptibility analysis, MIC data were first compiled in Microsoft Excel and subsequently analysed using R (version 4.x). Temporal trends in antifungal susceptibility of *C. auris* from 2021 to 2024 were analysed using interval-censored normal regression to accommodate MIC values reported in categorical form, including left-censored observations (e.g. ‘< 0.125 µg/mL’). Minimum inhibitory concentration values were log^2^-transformed and modelled as interval-censored outcomes. Models were weighted by isolate counts to account for the distribution of MIC observations. Calendar year was included as a continuous predictor to estimate the mean annual change (β) in MIC for each antifungal agent. Minimum inhibitory concentration breakpoints were interpreted according to Centers for Disease Control and Prevention guidelines.^21^

### Ethical considerations

Ethical clearance to conduct this study was obtained from the Faculty of Health Sciences Research Ethics Committee, University of Pretoria (No. 435/2025). Data were obtained via the NHLS by acquiring NHLS Academic Affairs and Research Management System (AARMS) approval (PR2563330). The data were anonymised with a unique identifier replacing the patient names to maintain confidentiality.

## Results

**FIGURE 1:**
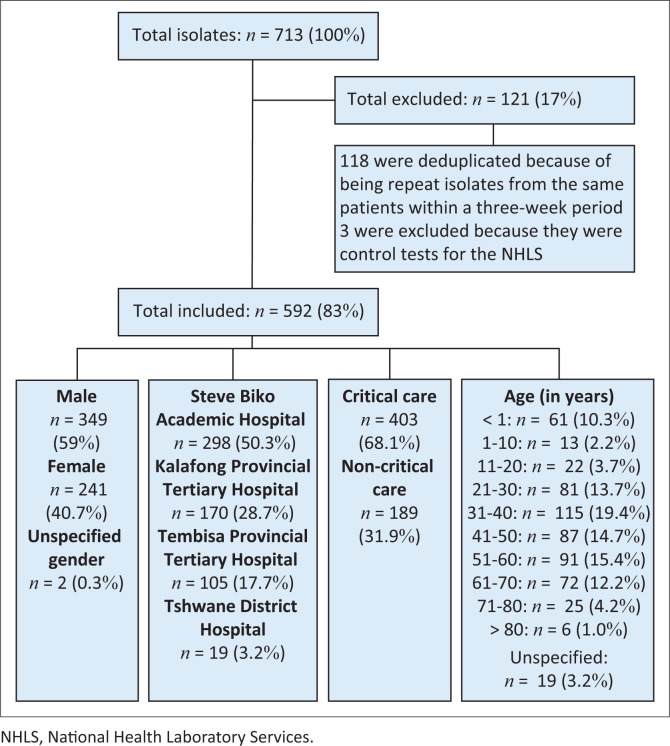
Flow diagram of *Candidozyma auris* isolates meeting inclusion criteria and their subdivision by gender, hospital, ward type, and age for the period 2021–2024 (*N* = 592).

Across the 4-year study period, a total of 713 *C. auris* isolates were identified, with 121 being excluded, while 592 were included, as shown in Figure 1. Of these, 349 (59%) originated from male patients and 241 (40.7%) from female patients, while the gender of two patients (0.3%) was not recorded (Figure 1).

A total of 403 isolates (68.1%) were recovered from patients admitted to critical care areas, including high care unit and intensive care unit (Figure 1). Steve Biko Academic Hospital reported the most isolates with 298 (50.3%), followed by Kalafong Provincial Tertiary Hospital with 170 (29.7%). A full breakdown of hospitals and age ranges of patients is available in Figure 1. Blood cultures were the most frequent specimen source overall, accounting for 237 isolates (40.03%) across the study period. However, in 2023 and 2024, catheter tip and wound swab specimens became the most common sources, with 48 and 72 isolates, respectively. A full yearly breakdown of specimen source and invasiveness is presented (Table 1).

**TABLE 1:** Breakdown of *Candidozyma auris* isolates from the greater Pretoria region (2021–2024) by invasive and non-invasive sources (*N* = 592).

Specimen source	Number of isolates by year								Total
	2021		2022		2023		2024		
	*n*	%	*n*	%	*n*	%	*n*	%	
**Invasive sources**									
Blood culture	77	-	50	-	41	-	69	-	237
Aspirate abscess	0	-	0	-	2	-	2	-	4
Tissue	1	-	1	-	0	-	1	-	3
Sterile site fluid aspirate	1	-	3	-	0	-	10	-	14
Subtotal (Invasive)	79	56.8	54	44.6	43	32.8	82	40.8	258
**Non-invasive sources**									
Intravascular catheter	42	-	44	-	48	-	72	-	206
Catheter urine	10	-	16	-	21	-	30	-	77
Midstream urine	2	-	2	-	6	-	6	-	16
Unspecified urine	5	-	2	-	8	-	5	-	20
Pus swab	1	-	1	-	3	-	0	-	5
Tracheal aspirate	0	-	1	-	1	-	3	-	5
Ventriculoperitoneal shunt	0	-	1	-	1	-	0	-	2
Sputum culture	0	-	0	-	0	-	2	-	2
Subtotal (non-invasive)	60	43.2	67	55.4	88	67.2	118	58.7	333
**Unspecified**	0	0	0	0	0	0	1	0.5	1

**Grand total**	**139**	**-**	**121**	**-**	**131**	**-**	**201**	**-**	**592**

Note: Invasive sources include isolates from normally sterile body sites (e.g. blood, Cerebrospinal fluid [CSF], tissue, and aspirates). Non-invasive sources include isolates from non-sterile sites such as skin, urine, respiratory specimens, and surface swabs.

CSF, Cerebrospinal fluid.

The proportion of invasive isolates declined from 56.8% in 2021 to 32.8% in 2023, before increasing to 40.8% in 2024, as shown in Table 1. A chi-square test of the yearly distribution of invasive and non-invasive isolates produced the following χ^2^ = 16.68; *p* = 0.0008. Linear regression of the proportion of invasive isolates over time produced a negative slope (β = −0.059 per year; *p* = 0.23).

Multiple antifungal agents were tested for susceptibility against *C. auris*, including fluconazole, voriconazole, amphotericin B, micafungin and caspofungin.

In total, 524 isolates were tested for fluconazole susceptibility. In 2022, 146 out of 147 (99.31%) isolates tested were resistant to fluconazole. Voriconazole susceptibility was also assessed during the same year; however, the absence of established clinical breakpoints resulted in variable interpretation of susceptibility. From 2023 onward, routine azole susceptibility reporting for *C. auris* was discontinued.

For amphotericin B, MIC distributions are shown (Table 2). The median MIC decreased from 1.0 µg/mL in 2021 to 0.5 µg/mL in 2024. Using a resistance breakpoint of ≥ 2 µg/mL, resistant isolates were limited, declining from two cases (1.4%) in 2021 to none in 2024.^21^ Interval-censored normal regression produced a slope of β = −0.059 per year (95% confidence interval [CI]: −0.106 to −0.013; *p* = 0.012), equivalent to an estimated 4% annual reduction in MIC values.

**TABLE 2:** Susceptibility of *Candidozyma auris* isolates from the greater Pretoria region to antifungal agents (2021–2024) using Sensitire YeastOne (*N* = 592).

MIC range (µg/mL)	Number of isolates by year				Interpretation
	2021	2022	2023	2024	
**Amphotericin B**					
0.064	0	3	0	0	S
0.25	8	8	5	9	S
0.5	60	41	65	83	S
1	80	77	63	67	S
2	2	1	1	0	R
**Micafungin**					
< 0.125	28	28	55	94	S
0.125	34	56	65	47	S
0.25	1	3	3	5	S
0.5	1	2	2	0	S
1	1	0	1	0	S
2	0	0	1	0	S
8	0	0	0	1	R
**Caspofungin**					
< 0.25	52	102	92	125	S
0.25	16	12	33	18	S
0.5	3	2	5	0	S
1	1	0	1	0	S
2	1	0	0	0	R
4	0	0	1	0	R
8	0	0	0	1	R

Note: Interpretation of MIC range (µg/mL) for amphotericin B: S (< 2), R (≥ 2). Interpretation of MIC range (µg/mL) for micafungin: S (< 4), R (≥ 4). Interpretation of MIC range (µg/mL) for caspofungin: S (< 2), R (≥ 2). Interpreted per the CDC guideline.^21^

MIC, minimum inhibitory concentration; S, Susceptible; R, Resistant; CDC, Centers for Disease Control and Prevention.

Micafungin MIC values are presented (Table 2). The median MIC remained 0.125 µg/mL from 2021 to 2023 and decreased to 0.0625 µg/mL in 2024. Using a resistance breakpoint of ≥ 4 µg/mL, one isolate (0.7%) in 2024 was classified as resistant.^21^ Interval-censored regression yielded a slope of β = −0.081 per year (95% CI −0.153 to −0.010; *p* = 0.026), corresponding to an estimated 5.5% annual reduction in MIC values. The single micafungin-resistant isolate in 2024 also showed resistance to caspofungin.

Caspofungin MIC values are shown (Table 2). The median MIC declined from 0.25 µg/mL in 2021 to 0.125 µg/mL in 2024. Using a resistance breakpoint of ≥ 2 µg/mL, resistant isolates were uncommon. A total of three cases were detected over the 4-year period, representing annual resistance rates 1.4% in 2021 (one case), 0.8% in 2023 (one case) and 0.5% in 2024 (one case).^21^ Interval-censored regression yielded a slope of β = −0.085 per year (95% CI: −0.204 to 0.034; *p* = 0.161.

## Discussion

This study provides a detailed overview of *C. auris* epidemiology and antifungal susceptibility patterns in the public health facilities of the greater Pretoria region of South Africa.

The total number of isolates increased annually, driven by a rise in non-invasive isolates, while the proportion of invasive isolates declined over time. This could reflect improved infection prevention measures rather than an expansion of clinical disease; however, we are not aware of any Infection Prevention and Control audits conducted in the hospitals. Bloodstream isolates accounted for 55.40% of all isolates in 2021 but declined to 34.33% by 2024, whereas isolates recovered from catheter tips and wound swabs increased over the same period from 30.22% to 35.82%. This redistribution suggests a gradual epidemiological transition from invasive infection towards colonisation and device-associated carriage. Similar shifts have been reported internationally, where heightened surveillance and early screening have reduced the proportion of *C. auris* bloodstream infections.^17^ The predominance of isolates from vascular access and skin-associated sites during the latter years of the study supports the hypothesis that indwelling medical devices serve as key reservoirs for ongoing transmission within healthcare facilities.^22^ These findings suggest that, although *C. auris* remains endemic in hospital environments, early identification of colonised patients can successfully mitigate the progression to invasive disease.

The majority of isolates in this study were cultured from patients admitted to critical care units. This finding aligns with the recognised predilection of *C. auris* for high-care hospital settings, where prolonged hospitalisation, invasive procedures, and broad-spectrum antimicrobial exposure facilitate both colonisation and transmission.^23^ Our study showed a slight predominance of the male gender, which is in keeping with other local studies.^5,6^

*Candidozyma auris* demonstrated near-complete resistance to fluconazole, with more than 99% of isolates classified as resistant. This finding is consistent with reports from both local and international studies, where similarly high rates of fluconazole resistance have been documented.^24,25,26^

Amphotericin B demonstrated a downward trend in MIC values over the study period. Although the total number of isolates increased, no resistant isolates were identified in 2024, in contrast to the low levels of resistance observed in previous years. Overall resistance remained uncommon, with only four isolates (0.7%) classified as resistant across the 4 years. This rate is slightly lower than that reported in earlier South African data from 2016 to 2017, where amphotericin B resistance was approximately 5% and below international estimates, which range between 8% and 35%.^26,27^

For the two echinocandins included in routine testing, micafungin showed a decline in mean MIC values over the study period, while caspofungin demonstrated a similar downward trend. Resistance remained uncommon, with one micafungin-resistant isolate (0.2%) and three caspofungin-resistant isolates (0.6%) detected. One specimen in 2024 displayed dual resistance to both echinocandins. These findings align with national and international reports, where echinocandins remain the most consistently active antifungal class against *C. auris*, although sporadic resistance has been reported at rates between 0% and 8%.^26,27^

One hypothesis for the reduction in antifungal MIC values is a shift in the dominant circulating lineage of *C. auris*. A previous South African study reported that changes in clade distribution over time may lead to lower aggregate MIC levels, even when laboratory testing methods remain unchanged.^28^ In addition, greater standardisation of susceptibility testing during the study period may have reduced variability in reported MIC values, as non-standardised methods have been shown to both over- and under-estimate amphotericin B MICs in *C. auris*.^14^

This study has several important limitations. Firstly, it was retrospective and relied entirely on laboratory surveillance data extracted from the NHLS CDW. As a result, detailed clinical information, including patient comorbidities, prior antifungal exposure, and treatment outcomes, was unavailable, preventing correlation of microbiological findings with clinical severity or mortality. Secondly, molecular characterisation of isolates was not performed; this limited the ability to confirm genetic mechanisms of resistance or track potential transmission clusters. In addition, the study covered public-sector facilities only, excluding private hospitals, and thus may not fully represent *C. auris* epidemiology across the wider Pretoria region. Furthermore, the isolates had to be referred to another laboratory for susceptibility testing as in-house testing was not available during the study period. This means that precise methodology, quality control and interpretive criteria are not known.

The strengths of this study include the following: The study included both invasive and non-invasive isolates across varying levels of hospital care, offering a comprehensive picture of the organism’s behaviour in routine clinical practice. A monitoring period of 4 years and broad inclusion criteria ensure that a good overview of isolates was obtained, and trends could be observed. All isolates were derived from the TAD Microbiology Department, ensuring direct relevance to the South African public healthcare context. Finally, the study population was unselected, reducing sampling bias and improving the generalisability of the findings.

## Conclusion

This 4-year surveillance study confirms that *C. auris* remains established within public-sector hospitals in Pretoria, with most isolates originating from critical care units. Over time, a shift was observed from invasive bloodstream infections towards non-invasive, device-associated specimens, with catheter-tip and wound isolates becoming more frequent than blood cultures. Antifungal susceptibility patterns remained stable, with echinocandins and amphotericin B retaining good activity despite consistently high fluconazole resistance. Ongoing molecular surveillance, together with antifungal stewardship, will be essential to detect emerging resistance and prevent onward transmission. Expanding surveillance to include the private healthcare sector will provide a more complete epidemiological picture and help to reduce the risk of drug-resistant *C. auris* becoming further established in the region.
